# The Effect of Lockdown Period during the COVID-19 Pandemic on Air Quality in Sydney Region, Australia

**DOI:** 10.3390/ijerph18073528

**Published:** 2021-03-29

**Authors:** Hiep Duc, David Salter, Merched Azzi, Ningbo Jiang, Loredana Warren, Sean Watt, Matthew Riley, Stephen White, Toan Trieu, Lisa Tzu-Chi Chang, Xavier Barthelemy, David Fuchs, Huynh Nguyen

**Affiliations:** 1 Department of Planning, Industry and Environment, P.O. Box 29, Lidcombe, NSW 2141, Australia; david.salter@environment.nsw.gov.au (D.S.); merched.azzi@environment.nsw.gov.au (M.A.); ningbo.jiang@environment.nsw.gov.au (N.J.); loredana.warren@environment.nsw.gov.au (L.W.); Sean.Watt@environment.nsw.gov.au (S.W.); Matthew.Riley@environment.nsw.gov.au (M.R.); Stephen.White@environment.nsw.gov.au (S.W.); toan.trieu@environment.nsw.gov.au (T.T.); LisaTzu-Chi.Chang@environment.nsw.gov.au (L.T.-C.C.); xavier.barthelemy@environment.nsw.gov.au (X.B.); david.fuchs@environment.nsw.gov.au (D.F.); hubert.nguyen@environment.nsw.gov.au (H.N.); 2 Environmental Quality, Atmospheric Science and Climate Change Research Group, Ton Duc Thang University, Ho Chi Minh City 700000, Vietnam; 3 Faculty of Environment and Labor Safety, Ton Duc Thang University, Ho Chi Minh City 700000, Vietnam; 4 Faculty of Engineering & Information Technology, University of Technology Sydney, Ultimo, NSW 2007, Australia

**Keywords:** COVID-19 lockdown, air quality, greater mtropolitan region of Sydney, WRF-CMAQ

## Abstract

In early 2020 from April to early June, the metropolitan area of Sydney as well as the rest of New South Wales (NSW, Australia) experienced a period of lockdown to prevent the spread of COVID-19 virus in the community. The effect of reducing anthropogenic activities including transportation had an impact on the urban environment in terms of air quality which is shown to have improved for a number of pollutants, such as Nitrogen Dioxides (NO_2_) and Carbon Monoxide (CO), based on monitoring data on the ground and from a satellite. In addition to primary pollutants CO and NO_x_ emitted from mobile sources, PM_2.5_ (primary and secondary) and secondary Ozone (O_3_) during the lockdown period will also be analyzed using both statistical methods on air quality data and the modelling method with emission and meteorological data input to an air quality model. By estimating the decrease in traffic volume in the Sydney region, the corresponding decrease in emission input to the Weather Research and Forecasting—Community Multiscale Air Quality Modelling System (WRF-CMAQ) air quality model is then used to estimate the effect of lockdown on the air quality especially CO, NO_2_, O_3_, and PM_2.5_ in the Greater Metropolitan Region (GMR) of Sydney. The results from both statistical and modelling methods show that NO_2_, CO, and PM_2.5_ levels decreased during the lockdown, but O_3_ instead increased. However, the change in the concentration levels are small considering the large reduction of ~30% in traffic volume.

## 1. Introduction

The COVID-19 pandemic in 2020 has caused death and economic misery in all countries of the world. The disease was named as Coronavirus Diseases 2019 (COVID-19) by WHO and is caused by a type of coronavirus strain called Severe Acute Respiratory Syndrome Coronavirus 2 (SARS-CoV-2) [1]. Although it is not as deadly in terms of death statistics as the 1918 H1N1 flu pandemic but it is unprecedented in the rapid transmission of viral agents from human to human around the world [2]. Starting in the city of Wuhan, China in late December, in a few months nearly all corners of the world was affected by the pandemic due to the highly contagious nature of the virus and the rapid transmission was also helped by the extensive global connectivity in our age as compared to previous times. Similar to the H1N1 virus of avian origin, the SARS-CoV-2 virus was thought to have its origin in a bat. In the first wave of the pandemic in early February to June 2020, many cities in the world were in the state of lockdown and many business activities were shut down in the effort to minimize the social contact and to contain the virus transmission.

Australia closed the border on 20 of March 2020 to all non-citizens and a week later most states in the country were in the state of lockdown. The state of NSW and the city of Sydney in particular was on lockdown for most of April and May with less restriction in June. The lockdown started on 16 March then a hard lockdown on 1 April 2020 and ease of lockdown from 15 May until 16 June when further easing with private homes could have up to 20 guests [3].

One of the consequences of the lockdown is the effect on the environment such as air quality in the urban area where anthropogenic activities were curtailed. Chen et al. (2020b) [4] have estimated the public health benefits due to a reduction in NO_2_ and PM_2.5_ during the COVID-19 lockdown: 8911 avoided NO_2_-related deaths and 3214 avoided PM_2.5_-related deaths from cardiovascular diseases, Chronic Obstructive Pulmonary Disease (COPD), and other diseases. Thus, lowering the ambient level of PM_2.5_ reduces the health burden in the exposed population.

Most researchers used the statistical method of control and treatment analysis to determine the change in air quality due to the COVID-19 lockdown, where the control is data from previous years and the treatment is data from the lockdown period in 2020. Rodríguez-Urregoa et al. (2020) [5] has reported, in their study of the effect of lockdown on the PM_2.5_ pollutant concentration in the 50 most polluted cities in the world, that PM_2.5_ levels were reduced in most of the cities compared to the levels before the lockdown. There are some cities where the PM_2.5_ levels increase during the lockdown but overall, the average reduction is 12% of PM_2.5_ concentration before the lockdown for these 50 cities. Chauhana and Singh (2020) [6], also reported the PM_2.5_ reduction in major cities around the world with the most pronounced reduction in Delhi and Mumbai in India. Similarly, Habibi et al. (2020) [7], using the same method of daily data of major cities around the world from the World Air Quality Index (WAQI) project for the January 2019–April 2020 period, has reported that NO_2_, CO, and PM_2.5_ levels reduced while O_3_ (Ozone) increased for most of the world’s major cities between February, March, and April 2020, as compared to the levels of the corresponding period in 2019. Berman and Ebisu [8] analyzed NO_2_ and PM_2.5_ air quality data in 2020 as compared to 2019 and reported that the levels of these pollutants decreased across the continental US. Brimblecombe et al. (2021) [3] also used the air quality data NO_2_ and PM_2.5_ during the lockdown period in 2020 and in the previous year in 2019 in Sydney to statically compare them and reported that the daily average levels of these pollutants decreased slightly but are statistically insignificant. Ming et al. (2020) [9] used air quality data in many cities across China and travel data from the Baidu website to study the air quality effect from the lockdown in China during the Lunar New Year and COVID-19 pandemic period. They reported a reduction of about 7 µg/m^3^ of PM_2.5_ during the pandemic lockdown across China. Chen et al. (2020a) [10] in their study on the impact of lockdown from 23 January to 2 February on air quality and PM_2.5_ composition in Shanghai reported a reduction in the concentrations of PM_2.5_, SO_2_, NO_x_, and CO but an increased level of O_3_ in Shanghai, as compared to those in the pre-lockdown period from 8 to 23 January. Similar results were obtained in the Chu et al. (2020) [11] study of other Chinese cities, in Huang et al. (2020) [12] of Hong Kong, Li and Tartarini (2020) [13] of Singapore, and Sharma et al. (2020) [14] of Indian cities. However, Donzelli et al. (2021) [15] in a study in Valencia, Spain has found that overall O_3_ levels decreased during the lockdown period, although this phenomenon was more closely related to weather conditions. The results from using the statistical method of control and treatment analysis as described above by various authors in different places are varying. Mostly a decrease in the levels of pollutants such as PM_2.5_, CO, and NO_2_ but an increase in the O_3_ levels were reported. However, there are some exceptions for cities that have different results for PM_2.5_ and O_3_ [5,15] or the change is insignificant [3]. These differences, at these excepted cities, could be due to the uncertainty in meteorology, which is at least equal to or larger than the change in emission due to the lockdown.

Other researchers used the diurnal analysis which showed in detail the change in air pollutant concentration at each hour of the day calculated as the average concentration from hourly data for each period under consideration. This method is usually used in conjunction with other methods to reveal more detail and provide a confirmation of the results from the statistical summary comparison method. Singh et al. (2020) [16] used the diurnal analysis, statistical summary control treatment method trend analysis, and probability distribution method to study the change in air quality over different regions in India including some megacities such as Delhi and found that, in general, a reduction in PM_2.5_, NO_2_, and CO while an increase in O_3_ occurred during the lockdown, especially in Delhi and higher populated centers. Shi and Brasseur (2020) [17] used only diurnal analysis with data from northern China including Wuhan and Beijing and reported that levels of PM_2.5_ and NO_2_ reduced significantly during the lockdown, while the mean O_3_ levels increased by a factor of 1.5–2. Jiang et al. (2021) [18] used the diurnal analysis with the summary statistics, probability distribution, and meteorological analysis to detect changes in air quality in many cities in China during the lockdown. The results showed that SO_2_, NO_2_, CO, PM_10_, and PM_2.5_ decreased, while the peak ozone increased in the afternoon during the lockdown.

Zangari et al. (2020) [19] analyzed the trend in air quality from January to May 2020 before and after the lockdown in New York City. They reported the improvement in PM_2.5_ by 36% and NO_2_ by 51% concentrations shortly after the shutdown took place. However, when the improvement trend in these pollutant concentrations were compared to those measured during the same period in 2015–2019, no significant difference between the years was found. For this reason, it is also important to consider the trend of air quality in the period before and after the lockdown in 2020 and compare it with those in the previous years to determine whether the improvement trend in 2020 is in fact due to the lockdown and not due to meteorological or seasonally emission variation.

Other studies used air quality models such as WRF-CMAQ to study the effect of lockdown on air quality. This method requires the emission inventory and meteorology host data as input to the model. Wang, Chen et al. (2020) [20] used the WRF-CMAQ model to predict the effect of COVID-19 lockdown in China on the PM_2.5_ concentration using two emission reduction scenarios during the lockdown and compared the prediction from these scenarios with that from the base case of the normal emission. They found that the PM_2.5_ level decreased by up to 20% in many cities in China. Wang, Zhang et al. (2021) [21] used the WRF-CMAQ simulation for the period before, during, and after the lockdown to estimate the change in air quality. The prediction from the reduced emission during the lockdown as compared with the normal emission in the pre- and post-lockdown showed an improvement in PM_2.5_, NO_2_, and SO_2_, while O_3_ had no obvious change. Marlier et al. (2020) [22] also used WRF-CMAQ to study the effect of meteorology on two simulation periods of 2019 (no lockdown) and of 2020 (lockdown in China) using same emission. By using observation data to compare the prediction in the two periods, they can explain the decline of PM_2.5_, NO_2_, and SO_2_ concentrations during the lockdown as due to the reductions in transportation and industrial pollution sources, but unfavorable meteorological conditions weaken the role of emission reduction. Zhao et al. (2020) [23] used WRF-CMAQ and applied a similar approach as [22] in the simulation of air quality in China using the same emission for two scenarios and observation data to isolate the impacts of meteorology and emission. The results were reported to have NO_2_ reduction, while O_3_ increased due to the emission change while PM_2.5_, CO, and SO_2_ were varied across many cities due to the strong meteorological influence. In addition to the WRF-CMAQ modelling tool, there are other air quality models such as the Tropospheric Chemistry Reanalysis Version 2 (TCR-2) used by Miyazaki et al. (2020) [24] with satellite chemical data assimilation products and emission reduction data to predict the air quality levels of NO_2_, O_3_, and PM_2.5_ during the lockdown in China.

The main reason for the reduction during the lockdown period is the reduced anthropogenic activities which cause the emission of air pollutants and hence, degrade air quality in the environment. The transportation sector, which is still much reliant on combustion type technology, is the main contributor to the emission of pollutants. Kaskaoutis et al. (2021) [25] have shown the reduction in Black Carbon (BC) concentration from fossil fuel combustion due to the lockdown period in Athens, Greece, and hence, a reduction in spectral scattering and absorption of aerosols.

**Figure 1:**
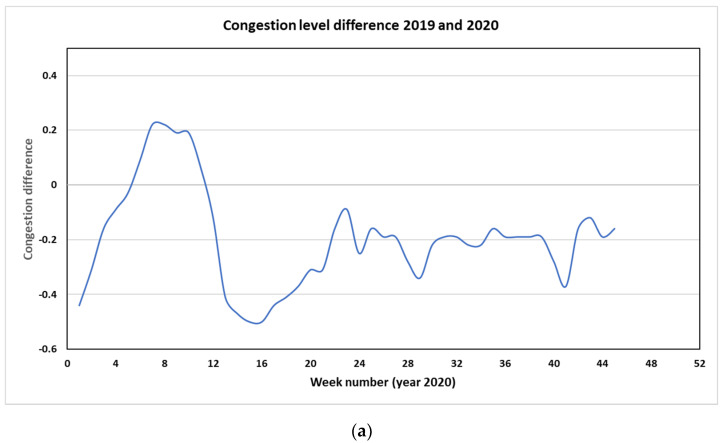

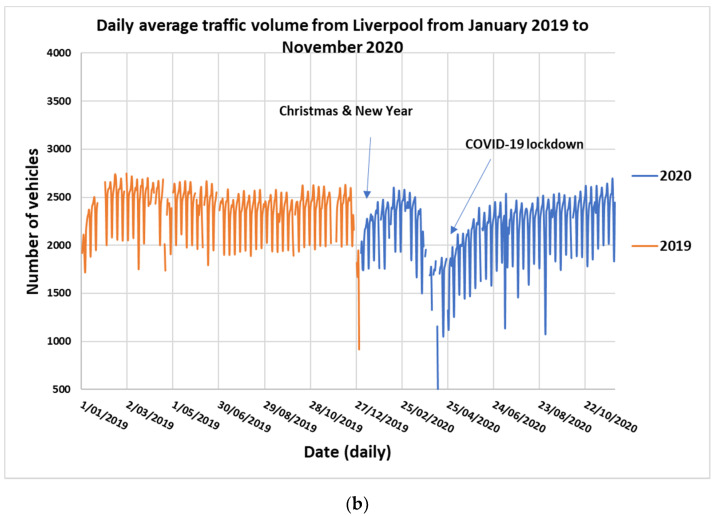
Congestion level relative difference between 2019 and 2020 (difference greater than the standard weekly congestion level in 2019) (**a**) (source: Tomtom traffic flow https://www.tomtom.com/en_gb/traffic-index/sydney-traffic/. Accessed on 19 March 2021) and daily number of vehicles on Liverpool Road as counted at a traffic site from January 2019 to November 2020 (**b**) (source: NSW Road and Maritime Services, RMS).

A significant reduction in traffic volume and hence, congestion on the Sydney road since the lockdown began in late March 2020 is shown in Figure 1a. The congestion level since then until the end of 2020 is less than that in the corresponding period in 2019. The congestion level is defined as the percentage of extra time that it takes to travel if there is no congestion. The congestion level in Sydney dropped to 40% compared to that in 2019 at the end of March 2020 with a maximum drop at about 50% in April then back to about 20% less than the 2019 level from May. This is expected as less traffic volume means less congestion in the metropolitan area. Figure 1b shows the daily average traffic volume from January 2019 to November 2020 on the Liverpool Road near Chullora monitoring station. A significant drop in traffic volume in April and May can be seen. The hourly traffic volume decreased from about 4000 vehicles to below 3000 in the first week of April, which was similar to the level in the first week of the Christmas and New Year holiday period.

With less emission from the motor vehicle, it is expected that the ambient levels of emitted species, such as NO_x_ and CO as well as for particulate matters such as PM_10_ and PM_2.5_, will reduce during the lockdown period. In Singapore, Li and Tartarini [13] in their study of COVID-19 mobility lockdown effect on CO, NO_2_, SO_2_, PM_2.5_, PM_10_, and O_3_ air quality have found that PM_10_, PM_2.5_, NO_2_, CO, and SO_2_ levels decreased by 23, 29, 54, 6, and 52%, respectively, whilst that of O_3_ increased by 18%. In addition, the trends of PM_2.5_ and NO_2_ were significantly correlated with mobility data.

In this study, we focus on the effect of lockdown on air quality in the Greater Metropolitan Region (GMR) of Sydney from April to June 2020, specifically on criteria pollutants CO, NO_2_, PM_2.5_, and O_3_. This study is different from some of the previous studies [5,6,7,15], in that we use a number of methods. We compare the air quality during the lockdown of 2020 with the air quality in the corresponding period of 2019 but also in previous years of 2016, 2017, and 2018 and a pooled ensemble of these previous years, as well as the diurnal analysis to control for the effect of meteorological variability. In addition, the detailed analysis on different sites besides the overall regional average, are conducted together with air quality modelling using the Weather Research Forecast—Community Multiscale Air Quality (WRF-CMAQ) model to understand the sensitivities of the air pollutants to change in the emission and meteorological conditions. There are other studies that also use multiple methods similar to ours, such as [16,18,22]. The consistent results from the methods applied were reported by these authors.

Our aim is to confirm the results of lockdown on CO, NO_2_, PM_2.5_, and O_3_ air quality in the Greater Metropolitan Region of Sydney and whether the methods give consistent results.

## 2. Data and Methodology

It is expected that the lockdown will improve air quality due to a substantial reduction in the mobile source emission. Traffic data from the NSW Road and Maritime Services (RMS) at many traffic counters located in the Greater Metropolitan Region (GMR) of Sydney are used to estimate the reduction in the amount traffic during the lockdown period as compared with traffic before the lockdown. The reduction in traffic gives a corresponding reduction in emission with the assumption that all the vehicles type distribution is the same over the GMR of Sydney. The estimated amount of emission reduction will then be used to perform a simulation study using the air quality model WRF-CMAQ to predict the change in air quality.

With air quality monitoring data available from the Department of Planning, Industry, and Environment (DPIE) air quality network in NSW, one of the methods used in this study is to compare the pollutant concentrations during the lockdown period in 2020 with those of the corresponding period in 2019 and 2018. Note that the assumption made implicitly here is that the meteorological conditions are essentially similar (autumn period April to June) and the emission (inventory) does not change much (except the mobile source reduction in the 2020 lockdown, otherwise all other sources are same). These two assumptions are not quite right, especially the meteorological component and hence, the dispersion of emitted pollutants, and we expect some variation at some sites. However, an analysis based on the above assumption can give us some indication of the comparative pollutant concentration and insights of air quality during the COVID-19 lockdown in 2020. This method of comparing the “control” group and “treatment” group was used by most authors such as [3,5,7,8,9,11,13,15]. However, as Zangari et al. (2020) [19] pointed out, recently the improvement trend in air quality before and after the lockdown could also be due to other factors. For this reason, we will also analyze the trend of air pollutants before and after the lockdown in 2020 and compare it with those during the same period in 2015–2019 to determine whether the seasonal variation in meteorology and emission can influence the results. A simple linear trend is fitted to the data using the Generalized Linear Model (GLM) in R with time as an independent variable.

*y_i_* = *a* + *bt_i_* + ε_i_

where *y_i_* is the pollutant concentration, *t_i_* is the time variable, ε_i_ is the error residual, and a, b is the intercept and coefficient, respectively.

In addition to the data-driven method, air quality simulation during the lockdown period using WRF-CMAQ is also conducted to take into account the meteorological and emission variation. The motor vehicle emission as input to the model is reduced by a quantified emission amount based on traffic volume data to predict the air quality variables under the lockdown as compared with no emission reduction scenario. Using statistics from a number of real time traffic counters in the GMR of Sydney before the lockdown in 2019 and during the lockdown period, it is estimated that the overall reduction in traffic volume was about 30% during the lockdown period. The motor vehicle emission from the 2013 NSW EPA emission inventory is reduced by that corresponding amount and used in the air quality model to simulate the effect on air quality during the lockdown period.

**Figure 2:**
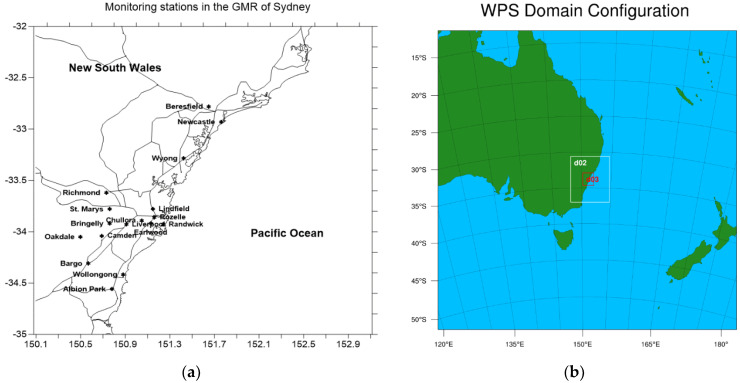
(**a**) Department of Planning, Industry, and Environment (DPIE) air quality monitoring stations and major roads; (**b**) the three-nested domains consisting of the outer domain (d01), inner domain (d02), and innermost domain (d03). The d02 domain covers most of New South Wales (NSW), while the d03 domain covers the greater metropolitan region (GMR) of Sydney.

In addition to the observed data from DPIE air quality monitoring stations in the GMR of Sydney (Figure 2a), data from the satellite observation and MERRA-2 global assimilation model before and during the lockdown period in NSW are also used to analyze the effect of lockdown on air quality in the GMR in particular and in NSW in general. The Ozone Monitoring Instrument (OMI) onboard Aura mission satellite measures criteria pollutants such as O_3_, NO_2_, SO_2_, and aerosols. With a number of retrieval products from OMI [26], the OMI-NASA retrieval product is used in this study to obtain the NO_2_ tropospheric column. Both MERRA-2 and OMI data are obtained from the NASA Giovanni online website (https://giovanni.gsfc.nasa.gov/. Accessed on 28 March 2021). Venter et al. (2020) [27] also used satellite data and ground-based monitoring data to assess the change in NO_2_ and PM during the COVID-19 pandemic and found a decrease in the levels of these pollutants globally.

The main pollutants, CO, NO_2_, PM_2.5_ (particulate matter less than 2.5 µm in diameter), and O_3_ (Ozone) are considered in this study. The anthropogenic emission sources that were affected by the lockdown are mostly on-road mobile sources such as motor vehicles, and to a lesser extent the aircraft emission and shipping emission. It is expected that the primary pollutants from these combustion sources, Carbon Monoxides (CO), Nitrogen Oxides (NO_x_), PM_2.5_, PM_10_, and Volatile Organic Compounds (VOC) such as toluene, benzene, and xylenes will be decreased. However, for ozone, a secondary pollutant, the effect can be complex and the level can increase or decrease depending on the location due to the interaction of meteorology driving the dispersion of primary pollutants NOx, VOCs, and the photochemical reactions forming the ozone levels across the domain. The statistical analysis such as box plot, regression method, and diurnal analysis is useful to assess the effect of lock down on air pollutant levels at different sites in the GMR. These statistical tools are available in the base R software (R.3.5.1) (The Comprehensive R Archive Network or CRAN. Available online: https://cran.r-project.org/. Accessed 27 March 2021) used in this study.

The COVID-19 lockdown period is an ideal case study of the source contribution of mobile sources on the ambient concentration of ozone and PM_2.5_ in the GMR. The air quality modelling tool such as WRF-CMAQ is used to simulate the ozone and PM_2.5_ levels in the GMR using emission data with and without the mobile source emission. Of the two precursors for ozone formation, the motor vehicle is the second largest source of NOx emission after power stations and VOC emission after biogenic emission in the GMR of Sydney. In the previous studies [28,29], the effect of motor vehicle emission is mostly pronounced in the south west Sydney where the maximum level of ozone is influenced by the emission of motor vehicle more than anywhere in the GMR. The prediction from the previous studies [28,29] can be tested and evaluated from the observation of ozone during the lockdown period in 2020.

The WRF-CMAQ modelling system developed by the US-EPA is a well-known air quality model used frequently around the world in many air quality studies [20,21,22,23]. This study uses the WRF-CMAQ model (version 5.3.1) (Community Modeling and Analysis System. Available online: https://www.cmascenter.org/cmaq/. Accessed 27 March 2021) based on CB6 (Carbon Bond 6 version 3 with an aerosol treatment of Secondary Organic Aerosol, cb6r3_ae7_aq) [30]. The GMR emission inventory data provided by the New South Wales Environment Protection Authority (EPA) [28,29] are used as anthropogenic emission input and the National Centers for Environmental Prediction (US NCEP) Global Reanalysis data as the meteorological driver [31]. Other emissions to the WRF-CMAQ include the global emission database EDGAR [32] for emission outside the GMR, the biogenic emission based on the MEGAN biogenic model, the marine aerosol (sea salt) and soil dust emission as provided with the CMAQ model [30]. To simulate the effect of COVID-19 lockdown, the motor vehicle emission is reduced by 30% on average as observed from the traffic count pattern at a number of traffic counter sites in the GMR.

The simulation domain configuration for the WRF-CMAQ run is a three-nested domain with the outer domain (d01) covering much of Eastern Australia at the resolution of 12 × 12 km. The inner domain (d02) is at 4 × 4 km resolution and covers most of NSW, while the innermost domain (d03) is at 1 × 1 km resolution and covers the Greater Metropolitan Region (GMR) of Sydney. Figure 2b shows the three-nested domain used for the WRF-CMAQ study simulation. The initial and boundary conditions for meteorology is from NCEP Final Reanalysis data [31] and the chemical species from the global model output CAM_chem (Community Atmosphere Model—Chemistry) [33] is available for download from NCAR (https://www.acom.ucar.edu/cam-chem/cam-chem.shtml Accessed on 27 March 2021).

Table 1 summarizes the methods, analysis, and data used in this study and in some of other studies from the literature to determine the effect of COVID-19 lockdown on air quality in various parts of the world. Other methods to predict the effect of different lockdown scenarios on air quality have also been used, such as the data-driven artificial neuron network (ANN) used recently by [34], as applied to the lockdown in Brazil, South America. However, this ANN method is not considered in this study. It is of interest to evaluate whether all the methods will produce consistent results which give us confidence in the results of the COVID-19 lockdown on air quality in the greater metropolitan region of Sydney.

## 3. Results

**Figure 3:**
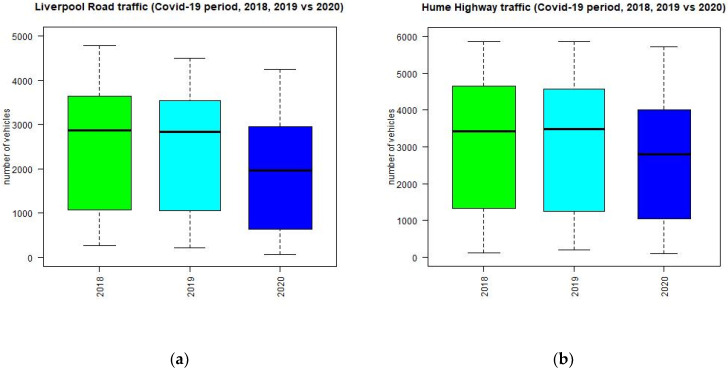

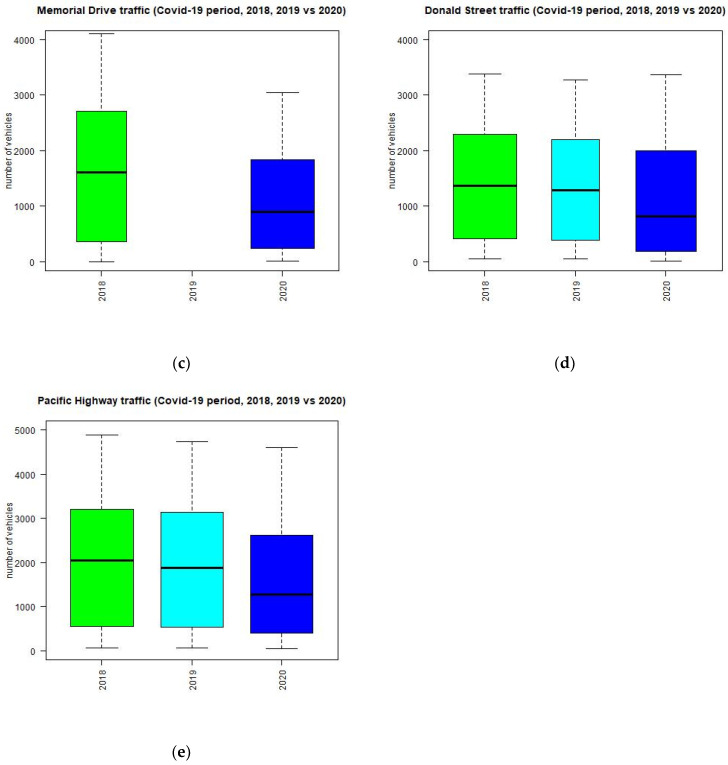
Boxplot of traffic volume per hour in 2018, 2019, and 2020 during the period of April to June at Liverpool Road (**a**), Hume Highway (**b**) in Sydney, Memorial Drive in Wollongong (**c**) and Donald Street (**d**), Pacific Highway (**e**) in Newcastle.

The traffic volume during the period from April to June 2020 at traffic sites is reduced significantly in 2020 compared to those in 2018 and 2019, as shown in Figure 3. The distribution of hourly traffic in 2018 and 2019 is nearly identical for sites shown in Figure 3, while the distribution in 2020 is different and has a lower median and maximum value than those in 2018 and 2019. Table 2 shows the location of some of the traffic counter sites used in the study. The sites are located on the main arterial roads and highways in the GMR of Sydney.

**Figure 4:**
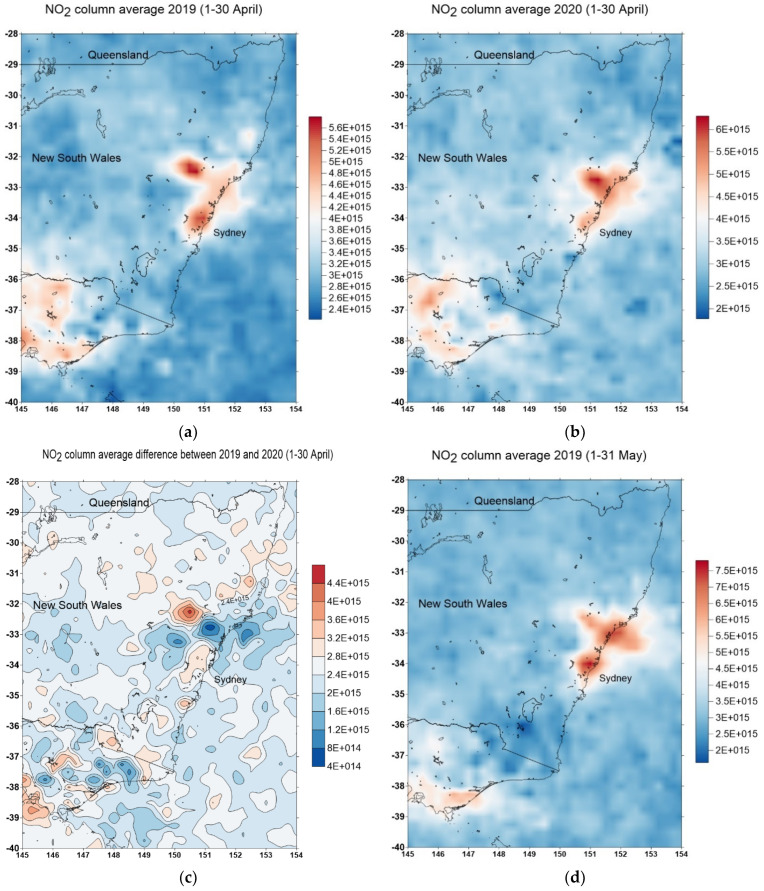

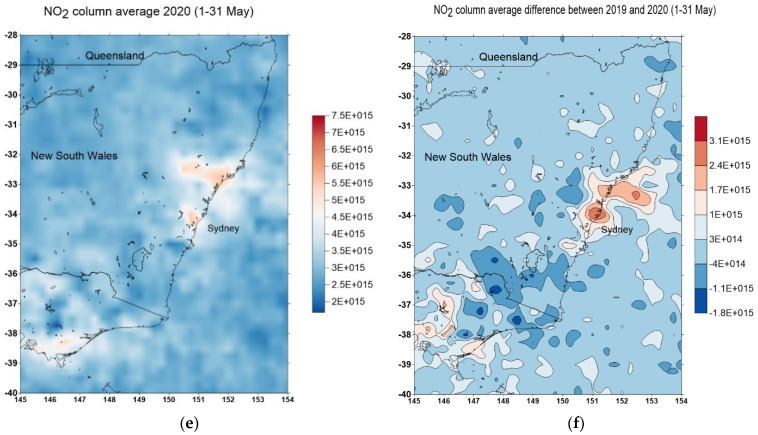
Time averaged map of NO_2_ tropospheric column (30% cloud screened) daily 0.25° [OMI OMNO2d v003] 1/cm^2^ in April 2019 (**a**), April 2020 (**b**), and the difference in April 2019 and 2010 (**c**). Similarly (**d**–**f**) are for May 2019 and 2020. The unit is in molecules/cm^2^ (data source: NASA Giovanni online https://giovanni.gsfc.nasa.gov/. Accessed on 27 March 2021).

The spatial extent of the effect on air quality can be seen from the OMI satellite measurement of column tropospheric NO_2_. Figure 4 shows the NO_2_ column tropospheric average value for April 2019, (a) April 2020, (b) and the difference of NO_2_ tropospheric column value in April 2019 and 2020 (c), while Figure 4d–f similarly shows the value for May 2019 and 2020. The most distinct reduction in NO_2_ is in May 2020.

**Figure 5:**
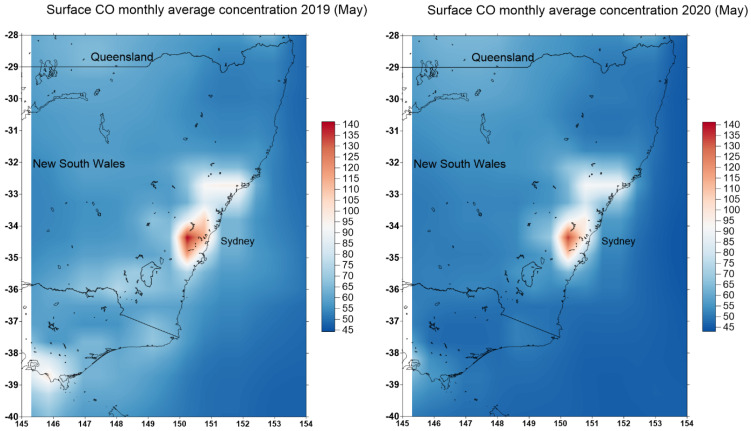

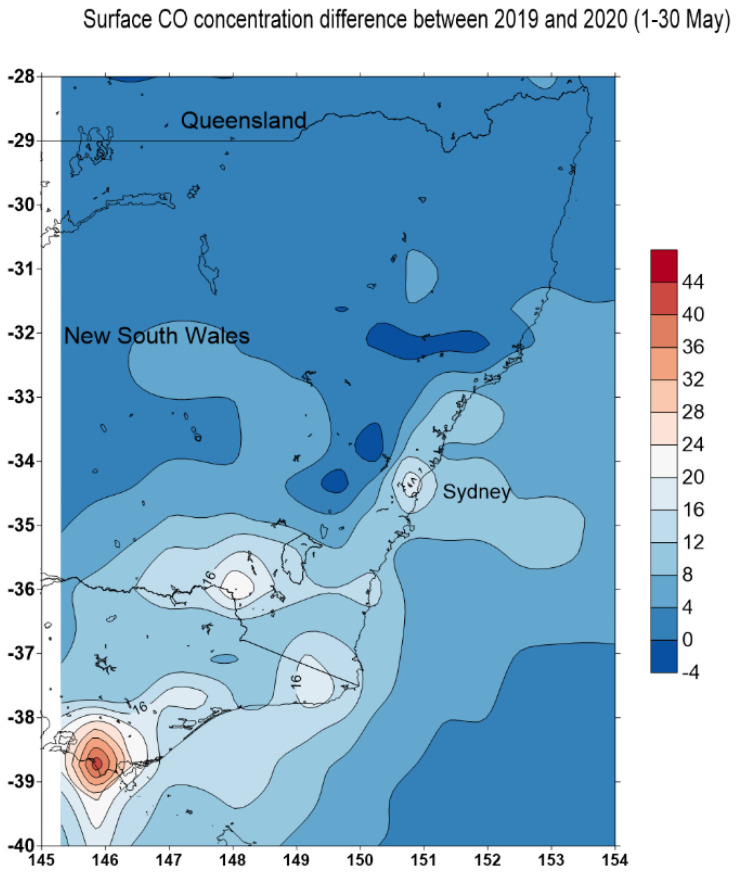
Monthly averaged map of CO surface concentration (ENSEMBLE) monthly 0.5 × 0.625° [MERRA-2 Model M2TMNXCHM v5.12.4]. The unit is in parts per billion volume (PPBV) (data source: NASA Giovanni online https://giovanni.gsfc.nasa.gov/. Accessed on 27 March 2021).

The CO observation from MOPITT and AIRS instrument on the satellites are of course the resolution at 1° (as compared to the OMI NO_2_ instrument at 0.25° resolution) and hence, are of limited values. However, the MERRA-2 global model provides better spatial resolution at 0.5 × 0.625° of the surface CO concentration. Figure 5 shows the predicted monthly CO surface concentrations in May 2019 and 2020. The surface CO concentration is reduced in the GMR. The largest reduction is in the metropolitan Melbourne in the state of Victoria.

### 3.1. Analysis of Air Quality Monitoring Data over the Whole GMR

Data from each monitoring site in the GMR are aggregated together to provide an overall status of the air quality over the whole region.

**Figure 6:**
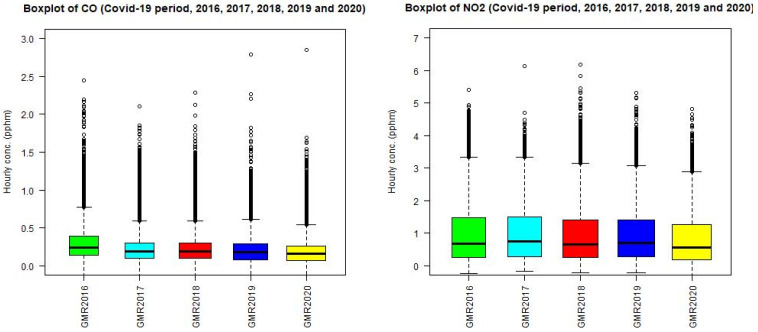

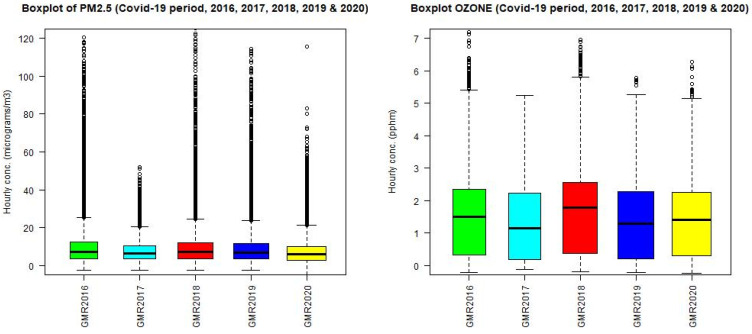
Boxplot of NO_2_, CO, PM_2.5_, and O_3_ for the period from April to June in 2017, 2018, 2019, and 2020 over all the sites in the GMR.

From the boxplot of NO_2_, CO, PM_2.5_, and O_3_ for the April to June period in 2016, 2017, 2018, 2019, and 2020 over all the sites in the GMR as shown in Figure 6, the median values of NO_2_, CO, and PM_2.5_ in 2020 are lower than those in 2017, 2018, and 2019. Table 3 summarizes the change in the median, mean, and maximum value of hourly data for the period from April to June for each of those years.

The change in NO_2_ median value in the 2020 lockdown as compared to the average mean value of previous years (2017, 2018, and 2019) is −18%. The figures for CO and PM_2.5_ are −13% and −13%, while for O_3_ is +1.5%. If a comparison is made between 2020 and 2019, the figures for NO_2_, CO, PM_2.5_, and O_3_ are −18%, −10%, −12%, and +10%, respectively.

For ozone, 2018 is a particularly warm year in the period of April to June compared to other years with the mean, median, and maximum ozone concentrations higher than those of 2017, 2019, and 2020. For this reason, if compared to 2018, the ozone level during the lockdown period in 2020 is still less than that in 2018, while for other years (2019 and 2017) the level in the 2020 lockdown is higher.

### 3.2. Analysis of Air Quality Monitoring Data from Monitoring Sites

**Figure 7:**
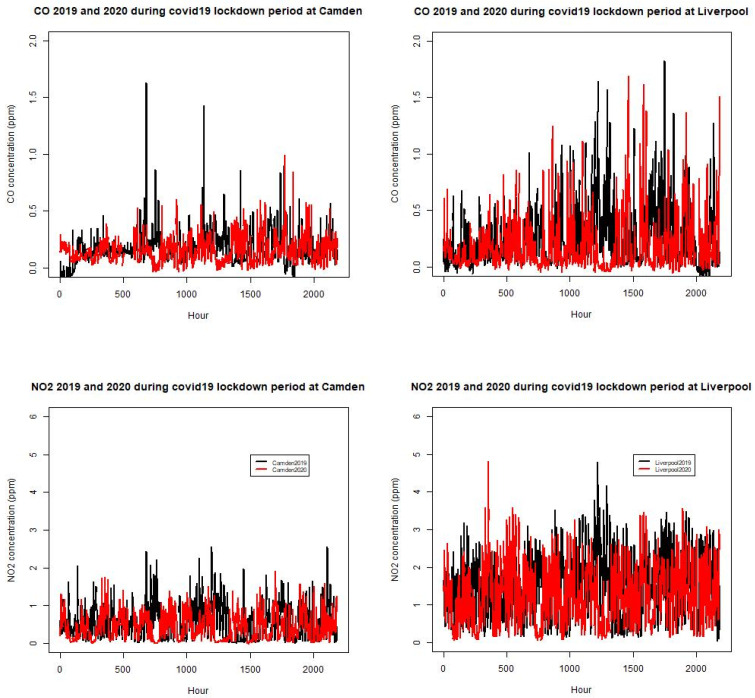
Time series of CO and NO_2_ at Camden and Liverpool for 2019 and 2020 during the April to June period (red as 2020 series, black as 2019 series).

The air quality monitoring station data at each site in the Sydney region provides valuable evidence for the understanding of the effect of lockdown on some air pollutants such as NO_2_, CO, O_3_, and PM_2.5_ in some sub-regions of the GMR. The time series of NO_2_ and CO at a number of sites (Camden and Liverpool in south west Sydney) for 2019 and 2020 in April to June are shown in Figure 7. Summary statistics from the pollutant time series allows us to compare the levels between the two considered periods.

**Figure 8:**
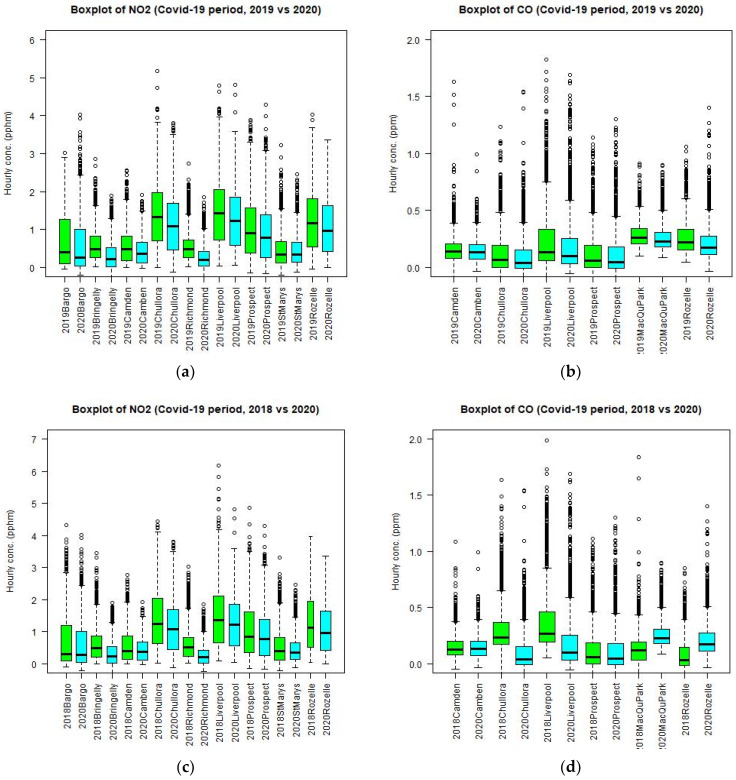

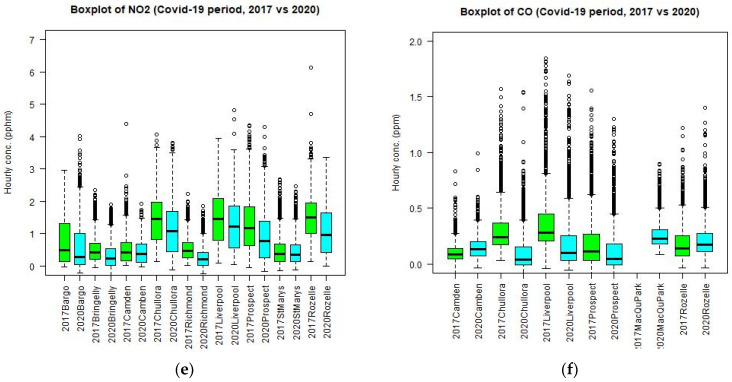
Boxplot of NO_2_ and CO at different sites in the GMR in 2019 and 2020 side by side for the period from April to June (**a**) and (**b**). Boxplot of NO_2_ and CO in 2018 and 2020 side by side (**c**,**d**). Additionally, in 2017 and 2020 side by side (**e**,**f**).

A summary of the time series can be represented by using the box plots. Figure 8 shows the box plots of NO_2_ and CO at different sites in the GMR for 2019 and 2020 for the period from April to June. It is clear that there is a decrease of NO_2_ and CO in median values at all the sites in the GMR.

**Figure 9:**
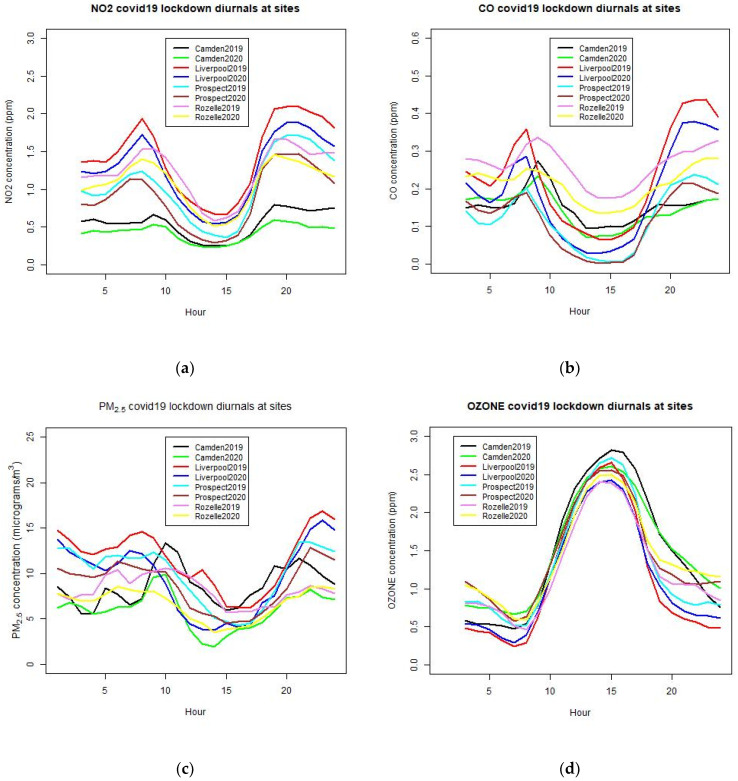
Diurnal patterns of NO_2_ (**a**), CO (**b**), PM_2.5_ (**c**), and O_3_ (**d**) for 2019 and 2020 from April to June at different sites in the GMR.

A more detailed analysis using average diurnal patterns of these pollutants for the lockdown period (April to June) also shows a decrease in the concentration of NO_2_ and CO during the daytime, as shown in Figure 9. The diurnal analysis provides finer details of the average change in air pollutant concentration at each hour of the day. This type of analysis has been used by many authors, such as [16,17,18], to detect the change in air pollutant concentration due to the COVID-19 lockdown. The CO diurnal (average of all the hourly data for the COVID-19 lockdown period at each hour of the day) for 24 h at a number of sites shows a typical pattern with two peaks, one in the early morning and one in the early evening. For all the sites considered, the 2020 COVID-19 lockdown period as compared to 2019 of the same period shows a drop of CO for all the hours except from midnight to the early morning before 7 am at some sites (Camden and Prospect). At this time, the traffic emission is insignificant. However, for NO_2_ and PM_2.5_ a decrease in the concentration level at all sites for all hours is shown. For O_3_, increasing levels from 6 pm to about 8 am the next morning and decreasing levels during most of the daytime is shown. Overall, an increase in the median O_3_ level is shown but the peak ozone is decreased. This is due to less NOx at night, which resulted in less scavenging of O_3_ and hence, the level of O_3_ increases.

The results from the NO_2_, CO, PM_2.5_, and O_3_ diurnal analysis are consistent with those from the boxplot statistical analysis of the control and treatment method.

For PM_2.5_, the median and maximum level at nearly all sites (except at Richmond in north west Sydney) decreases during the lockdown period as compared to the 2019 and 2018 levels of the corresponding period. The difference in meteorological conditions or emission in 2019 (or 2017, 2018) and 2020 can influence the results of comparison but the general trend is that the levels of PM_2.5_ decrease during the lockdown in 2020 as compared with those if the lockdown did not occur.

**Figure 10:**
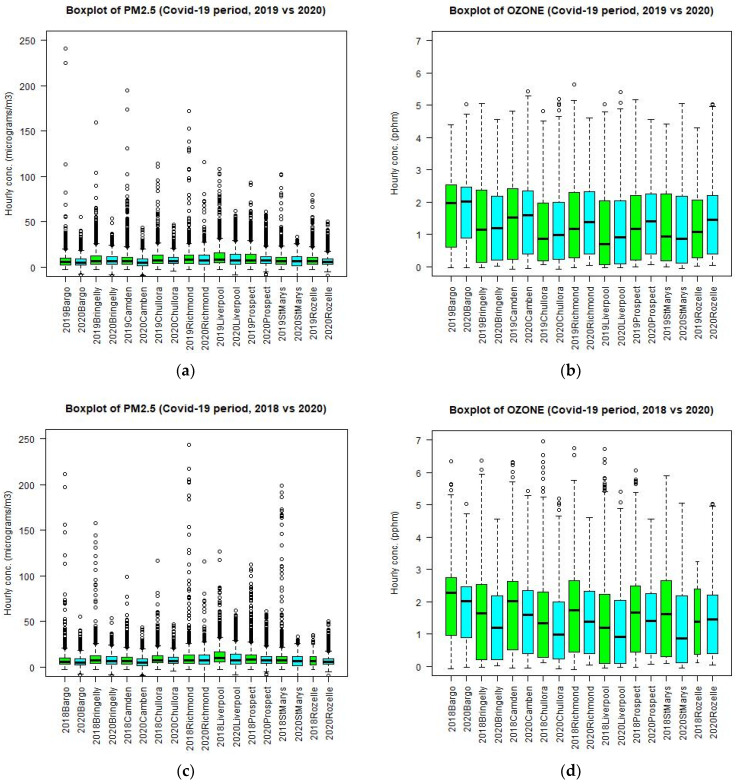

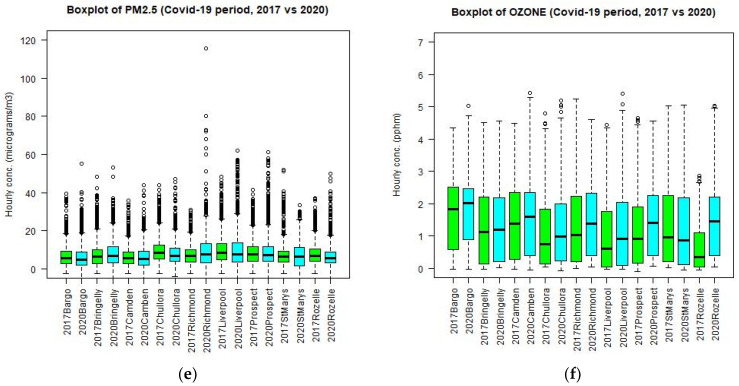
Boxplot of PM_2.5_ and ozone at different sites in the GMR in 2019 and 2020 side by side for the period from April to June (**a**) and (**b**). Boxplot of PM_2.5_ and ozone in 2018 and 2020 side by side (**c**,**d**). Additionally, in 2017 and 2020 side by side (**e**,**f**).

However, the effect of lockdown on the ozone concentration is different from those on CO, NO_2_, and PM_2.5_ levels. The box plots of ozone levels at different sites in 2019 and 2020 are shown in Figure 10. The ozone median level at most sites (except St Marys) in fact increases during the lockdown period as compared to the levels in 2019 (Figure 10b) but the maximum level decreases at some sites (Bringelly, Richmond, Prospect) and increases at other sites (Bargo, Camden, Liverpool, Chullora, St Marys, Rozelle). This inconsistency in the trend of ozone levels between 2019 and 2020 at different sites could be due to different meteorological conditions, as well as the photochemistry mechanism in the April to June period for these 2 years. For this reason, the comparison of ozone during the lockdown period in 2020 with those in 2017 and 2018 data is also performed. The results for 2018 are more consistent. Both median and maximum ozone levels in 2020 (Figure 10d) decrease at nearly all sites as compared to those levels in 2018 for the corresponding period of April to June. However, as compared with the 2017 corresponding period, the ozone levels in 2020 are higher than those in 2017. It is noted that the daily maximum temperature in 2018 for April to June is on average higher than those in 2017, 2019, and 2020. As the ozone level is highly correlated with the temperature, which is acting as a proxy for the photolysis rate, it is expected that the average ozone levels in 2018 over the GMR are higher than those of 2017, 2019, and 2020.

**Figure 11:**
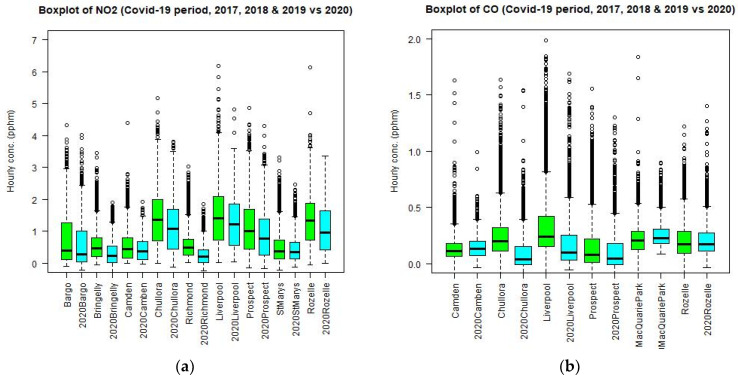

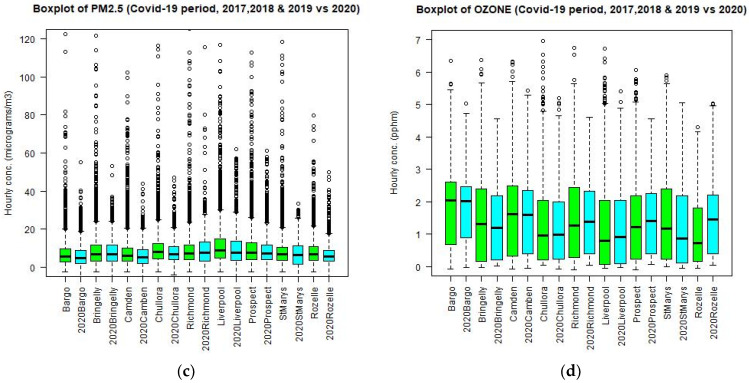
Boxplot of NO_2_ (**a**), CO (**b**), PM_2.5_ (**c**), and O_3_ (**d**) at different sites in the GMR based on pooled 2017, 2018, and 2019 data and those based on 2020 side by side for the period from April to June.

If we combine the previous years (2017, 2018, and 2019) of hourly values of CO, NO_2_, PM_2.5_, and O_3_ and compare them with the 2020 values, we can reduce the effect of interannual meteorological variability and emission uncertainty when a comparison is made against the pooled values of 3 years. Figure 11 shows the boxplots of CO, NO_2_, PM_2.5_, and O_3_ of the pooled values of 2017, 2018, and 2019 as compared with those of 2020. The results are similar to previous ones with the NO_2_ median and maximum levels decreasing at all sites and CO levels decreasing at most sites. For PM_2.5_, the median levels decrease at most sites and for O_3_ the median levels mostly increase but the changes for these two pollutants are small.

Putting all site measurements together for the whole GMR of Sydney for each year from 2016 to 2020, the results, as shown in Figure 6, show that for all pollutants (NO_2_, CO, PM_2.5_, and O_3_), the COVID-19 lockdown period of 2020 in the GMR caused less CO, NO_2_, and PM_2.5_ and increase in O_3_ as compared to 2019. However, if other years are taken into consideration, the change is not noticeable or significant when looking over the interannual trend.

### 3.3. Trend Analysis

**Figure 12:**
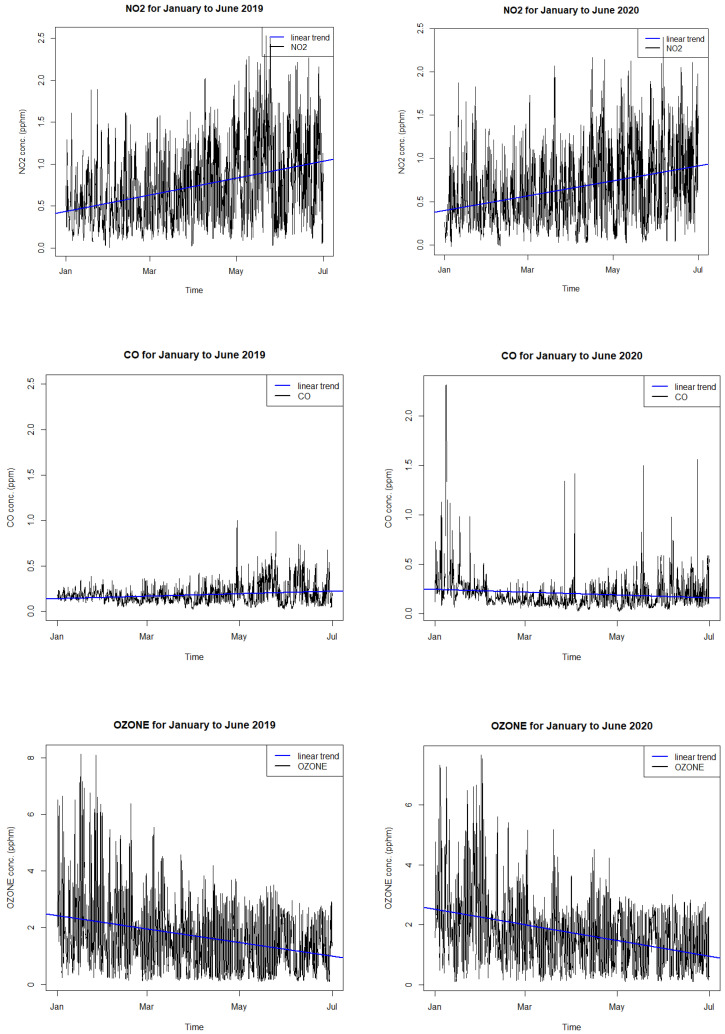

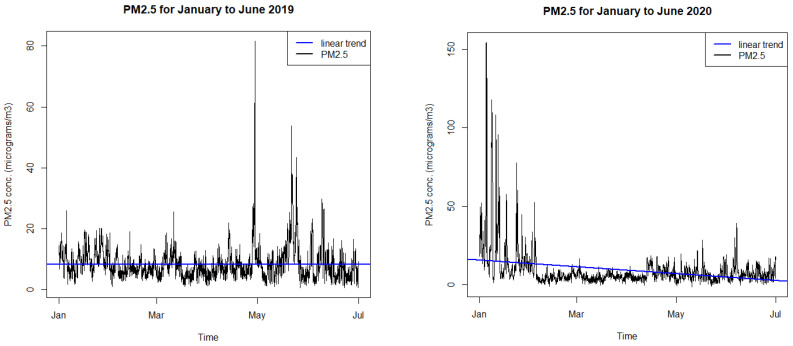
Average hourly concentration of all monitoring sites of NO_2_, CO, O_3_, and PM_2.5_ from the January to June period in 2019 and 2020 and the linear trend using the generalized linear model (GLM).

The trend of air quality in 2020 before and after the lockdown period as compared with trends in previous years during the same period has been used by [19]. We analyze the trends of average hour concentration of all Sydney monitoring sites for NO_2_, CO, PM_2.5_, and O_3_ from the January to June period in 2020, which covers the time before and after the lockdown period and the trends of these pollutants in 2019. As shown in Figure 12, it can be seen that the trends for NO_2_ in 2019 and 2020 are both increasing, but the NO_2_ level in April and May 2020 is lower than that in 2019. There is no indication that the lockdown period in April and May caused a downward trend in NO_2_. The increasing trend is mainly due to the photochemical reaction change involving NO_x_, CO, O_3_, and Volatile Organic Compound (VOC) from austral summer (January-February) to autumn (March to May) and winter (June to August) when the temperature is decreasing. In January and early February 2020 before the lockdown period in April and May, the summer 2019–2020 wildfires caused PM_2.5_ and CO levels to be elevated in the Sydney region. Therefore, the downward trend of these pollutants is due to the decrease in emission from a natural source. As for the decreasing trend in O_3_, it is due to the seasonal change in meteorology when the temperature drops in the cooler weather of autumn and early winter. In addition, emission also changes seasonally such as solid fuel heating and temperature-dependent tail-piped and evaporative emission. Solely using the trend to detect the impact of lockdown on air quality is not reliable.

### 3.4. Meteorological Analysis

**Figure 13:**
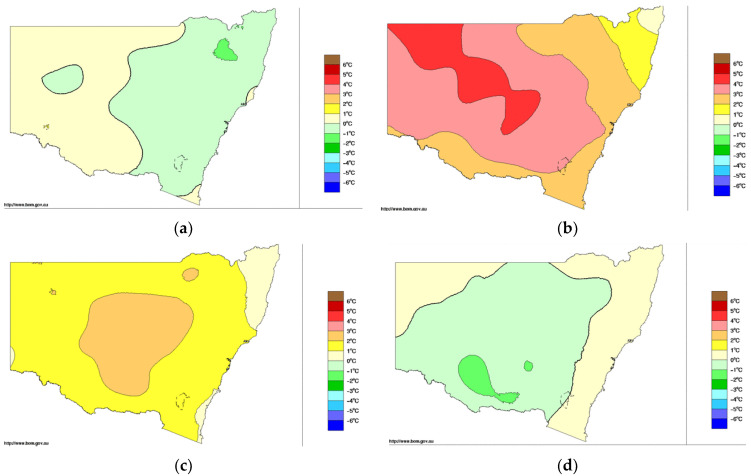
Mean temperature anomaly in NSW for April: (**a**) 2017, (**b**) 2018, (**c**) 2019, and (**d**) 2020. (Adapted with permission from http://www.bom.gov.au/jsp/awap/temp/archive.jsp?colour=colour&map=meananomr&year=2020r&month=4r&period=monthr&area=ns, accessed on 29 March 2021, under Creative Common License as at https://creativecommons.org/licenses/by/3.0/au/, accessed on 29 March 2021. Copyright 2020, Commonwealth of Australia, Bureau of Meteorology)

It is important to control the change in meteorology in the “control and treatment” method when comparing the air quality in the lockdown period in 2020 with those in the previous years of the same corresponding period. The assumption is that on average the meteorological conditions over the period from April to June is similar in 2020 and to those in previous years. However, as mentioned before, 2018 is a particularly hot year compared to others, the ozone level on average in 2018 is higher than those of other years. Figure 13 shows the anomaly of mean temperature across NSW in different years as compared to the average over the standard reference period of 1961–1990.

From Figure 13, the year of 2020 is a cooler year in April as compared to the same month in 2019 and 2018, but is about the same as in April 2017. Note that 2018 is a hot year in April. For May, the patterns are also similar to those in April with May 2020 being cooler than May in previous years. However, for June, the mean temperature anomaly in 2020 is similar to those in June of previous years. The hotter year in 2018 of April and May explains why the median ozone level in 2018 is higher than the corresponding level in 2020 of the COVID-19 lockdown (Figure 7), while the levels in 2017 and 2019 are lower than the COVID-19 2020 level (Figure 10b,f). In other words, the meteorological effect in 2028, causing a higher ozone in 2018 as compared with that in 2020, is stronger than the effect of the reduced emission in 2020 causing a higher ozone in 2020 as compared with 2018. This shows the importance of controlling the effect of meteorology in different years when the effect of reduced emission on air quality such as during the lockdown in 2020 is being investigated. The ozone formation process from photochemistry showed that, as the airshed is in a VOC-limited or light-limited regime, the reduction of NOx causes a higher ozone production. In addition, when the airshed is in the NOx-limited regime, a reduction of NOx causes a reduction of ozone. Most of the time in the Sydney region, the photochemistry processes are in the VOC-limited regime except during high ozone event days.

### 3.5. Air Quality Modelling of Emission Change during the Lockdown Period

**Figure 14:**
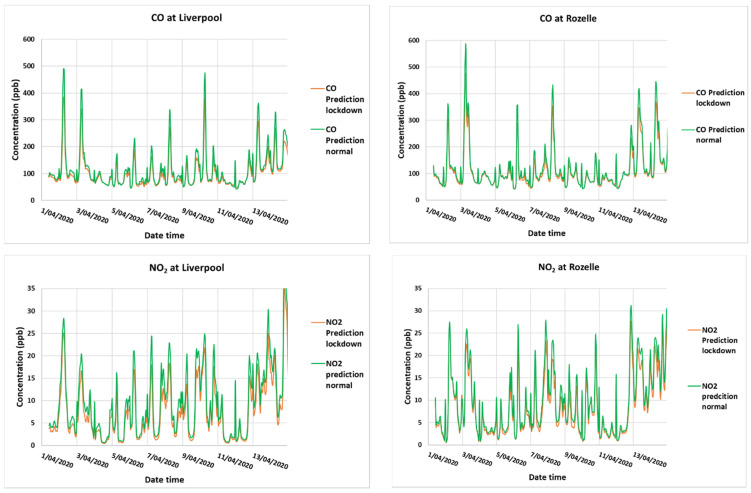

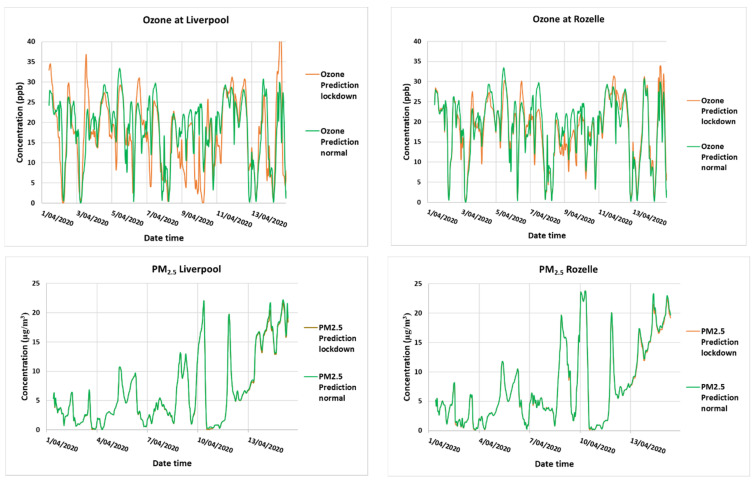
CO, NO_2_, O_3_, and PM_2.5_ prediction from 1 to 14 April 2020 at Liverpool and Rozelle during the lockdown with 30% less traffic emission and with normal traffic emission.

The simulation results from WRF-CMAQ 5.3.1 are obtained for the three domains d01, d02, and d03. As we focus on the Sydney metropolitan area, the inner most d03 domain (1 × 1 km) is used to extract the time series of CO, NO_2_, O_3_, and PM_2.5_ at each of the grid point nearest to the location of the monitoring sites from the results of simulation (1 to 14 April 2020). Simulation is based on emission before the lockdown (normal emission) and reduced emission during the lockdown. Figure 14 shows the time series of these pollutants from 1 to 14 April 2020 at Liverpool in the south west of Sydney and Rozelle in the Sydney center, as predicted from the WRF-CMAQ model for the above two scenarios.

**Figure A1:**
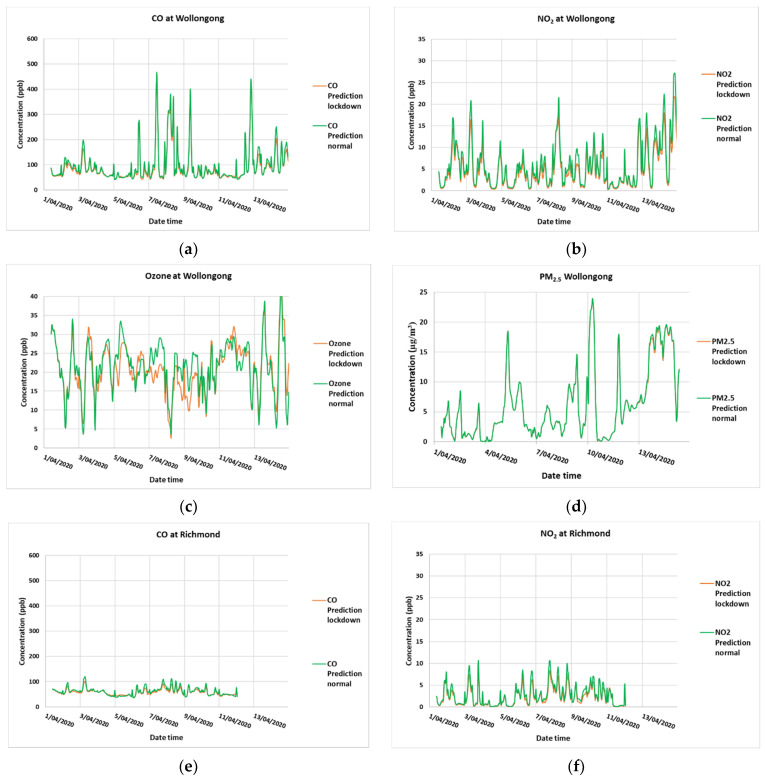

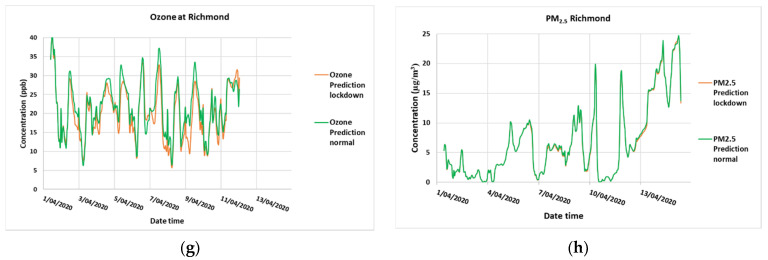
CO, NO_2_, O_3_, and PM_2.5_ prediction from 1 to 14 April 2020 at Wollongong (**a**–**d**) and Richmond (**e**–**h**) during the lockdown with 30% less traffic emission and with normal traffic emission.

**Figure A2:**
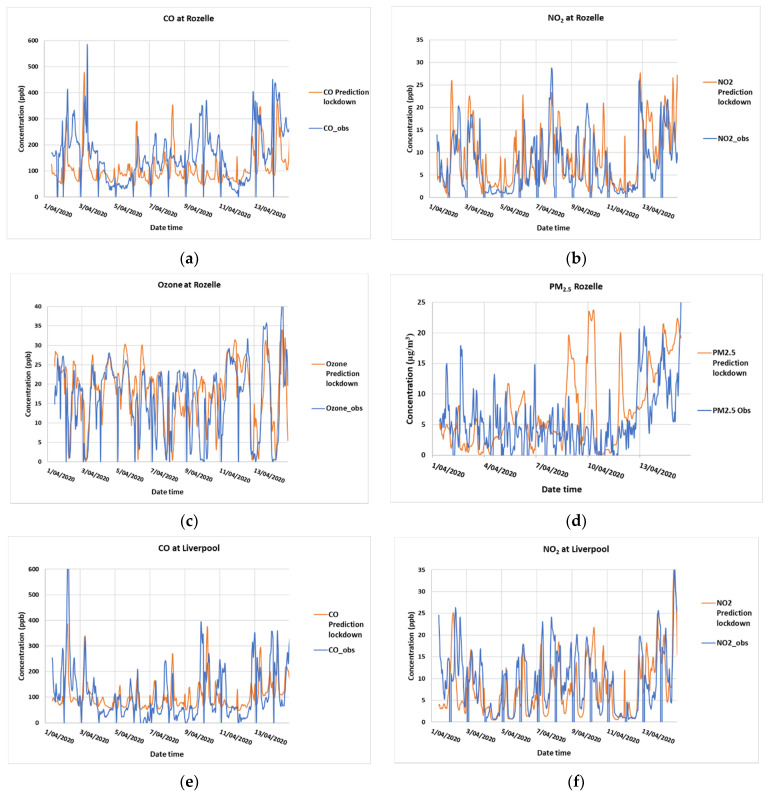

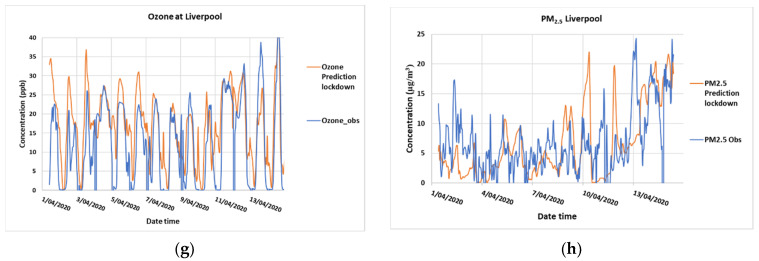
CO, NO_2_, O_3_, and PM_2.5_ prediction from 1 to 14 April 2020 at (**a**–**d**) Rozelle and (**e**–**h**) Liverpool during the lockdown, as compared with the observation. The predicted PM_2.5_ consists of the following subcomponents: PM25_EC (elemental carbon), PM25_OC (organic carbon), PM25_OM (organic matter), PM25_CL (chlorine), PM25_MG (manganese), PM25_NO3 (nitrate), and PM25_NH4 (ammonium) as defined in the CMAQ model.

Figure A1 of the Appendix A shows the results for other sites, Wollongong in the Illawarra south of Sydney and Richmond in the north west of Sydney, where the influence of traffic is much less than the sites of Liverpool and Rozelle. However, the results are similar. For CO and NO_2_, the decrease of the pollutant concentration during the lockdown period when the traffic emission is about 30% less than the normal traffic emission is predicted for all the sites in the GMR. The decrease is rather not significant. For O_3_, the daily peak values in 2020 most often increase during the period of simulation even though the increase is small. Figure A2 in the Appendix A shows that the prediction of CO, NO_2_, and O_3_ from the simulation using WRF-CMAQ for the lockdown period from 1 to 14 April 2020 corresponds well with the observation. For PM_2.5_, however, the soil dust component of PM_2.5_ from the dust emission module in CMAQ is overestimated. Removing this soil component improves the total PM_2.5_ predicted concentration as compared to the observation. A comparison of the predicted PM_2.5_ during the lockdown period and normal period shows that there is virtually no change in the predicted PM_2.5_ concentration with the decrease in PM_2.5_ (mainly Elemental Carbon, Organic Carbon, Organic Matter components) during the lockdown, which is too small to be detected.

## 4. Discussion

Of the four pollutants CO, NO_2_, PM_2.5_, and O_3_, when a comparison is made between the levels of those pollutants in 2020 and those of the previous years (2017, 2018, and 2019) only O_3_ did not show a decrease in the concentration consistently. Using monitoring data, the effect of the lockdown could be masked by different meteorological conditions and emissions in 2017, 2018, 2019, and 2020. Our results are consistent with the results from other studies in cities around the world such as [10] in Shanghai, [7] in 10 major world cities including Sydney and Perth in Australia, [11] in Chinese cities, [13] and in Singapore. Rodríguez-Urregoa et al. (2020) [5] compared the PM_2.5_ time series of a typical week during the lockdown with that of 1 week before the lockdown for the 50 most polluted cities in the world. They reported that most of the cities such as Dhaka, Delhi, Ulanbator, Colombo, Tashkent, Kuwait City, Tehran, and Beijing have the PM_2.5_ level significantly reduced from 40% highest (Delhi) to 8% (Beijing). However, other cities such as Kathmandu, Hanoi, Jakarta, Singapore, and Tokyo had an increasing trend of PM_2.5_ concentration by 11%. Their method of comparison using the 1-week PM_2.5_ time series data entering the lockdown and a typical week time series before the lockdown is rather short and does not take into account the meteorological and other variabilities and therefore cannot explain the reason for the change in the PM_2.5_ level as due to the emission reduction or due to the meteorological effect.

Habibi et al. (2020) [7] conducted a similar study using the 10 world major cities air quality data from WAQI for the February to April period. Sydney and Perth from Australia were included. The comparison was made between February to April 2020 and 2019. Sydney was reported to have a reduction in NO_2_, CO, and PM_2.5_ in February, March, and April but an increase in O_3_ in February and April. Their results correspond to ours using the March to June period. Sydney has the lowest reduction of NO_2_ emission among the 10 cities but the highest reduction of PM_2.5_ (−34.5%) in March to April. To account for the change in meteorology, temperature, and wind speed for the months of February, March and April in 2019 and 2020 were also considered. There are little changes in the monthly temperature median values of these parameters for Sydney. Our results of reduction in NO_2_, CO, and PM_2.5_ levels and increase in the O_3_ level during the April to June 2020 lockdown period correspond to their results, which are based on data from February to April. However, our results show the change is relatively small. Using air quality data in Sydney from April 2019 and 2020, Brimblecombe and Lai (2021) [3] compared the NO_2_, PM_2.5_, and O_3_ levels and reported that the decrease during the lockdown period of 2020, as compared with levels in 2019, is small and not significant. Their results correspond to those in our study.

The decreased level of PM_2.5_ during the lockdown period can be due to both the decrease of primary PM_2.5_ and the reduction of secondary inorganic aerosols from less nitrate formation. Chen et al. (2020a) [10] in their study of the impact of lockdown on the chemical composition of PM_2.5_ in Shanghai showed that the reduction in PM_2.5_ is attributed to the decreasing concentrations of primary aerosols and nitrate and the decreasing level of NO_x_ led to the increasing O_3_ and decreasing nitrate. They also found that as the proportion of nitrate in PM_2.5_ decreased the proportion of sulphate and oxygenated organic aerosols (OOA) increased and hence, inhibited the decrease of PM_2.5_ level further.

The increase in the median O_3_ level during the 2020 lockdown period is not unexpected as the ozone formation in the GMR is mostly in a VOC-limited (or light-limited) regime and hence, a reduction in NO_x_ (or NO_2_) will increase the rate of photochemistry reaction and hence, the ozone level. Wang, Wen et al. (2020) [35], using the machine learning method, also reported increasing levels of O_3_ during the 4-month lockdown period in six mega-cities in China. Chen et al. (2020a) [10] also reported that the O_3_ level increased in Shanghai during the lockdown period as a result of the decreasing NO_x_ level. The results from our study highlights the need to reduce the VOC emission, as well as the NO_x_ emission to improve the ozone level in the GMR since a reduction in NO_x_ is not necessary to improve the ozone. The previous source apportionment study to the ozone level in the Sydney Metropolitan Region [28] indicated that the motor vehicle emission has the largest influence on the maximum ozone level in the south west and west of Sydney and a reduction in the motor vehicle emission will improve the maximum ozone level in the GMR. Figure 11d shows that the maximum ozone level is improved during the 2020 lockdown period for sites in the south west of Sydney (Liverpool, Camden, Bargo, Bringelly, and StMarys) even though the median level increases at most sites.

To control the effect of meteorology, the air quality model such as WRF-CMAQ is used. In this way, the effect of lockdown due to the change in emission on air quality can be simulated. The results from the simulation for the first two weeks of the lockdown period in April confirmed the results using the statistical analysis of air quality monitoring data. The CO, NO_2_, and PM_2.5_ concentrations decreased, while O_3_ increased during the lockdown. However, the changes in those concentrations are not significant. Similarly, the simulation study using the WRF-CMAQ air quality model for the 2017, 2018, 2019, and 2020 April to June period with global meteorological data from these years with the same emission data input can allow us to find out the influence of meteorological conditions on the ozone level separately. This approach was used by [22,23] in their studies of the impact of COVID-19 response actions on air quality in China. It is noted that the soil dust emission in WRF-CMAQ 5.3.1 is overestimated. This overestimation was also observed by [36]. Removing this wind-blown dust (WBD) component improves the PM_2.5_ prediction as compared with the observation.

In our simulation using the air quality model for the COVID-19 lockdown period of April to June 2020 in Sydney, there is uncertainty in quantifying the changes in emission. The effect of lockdown had an impact not only on the motor vehicle emission, shipping, and aircraft emission but also can potentially increase the domestic sector emission and lead to a decrease of emission in the commercial sector. Therefore, the overall domestic and commercial emission can offset some of the transport sector emission reduction due to this trade-off. This study does not consider those changes in the emission in other sectors.

Using the trend method, Zangari et al. (2020) [19] found no significant difference in the trend of PM_2.5_ and NO_2_ in the period before and after the lockdown with those of the previous years during the same period. This result of no improvement in air quality from the COVID-19 lockdown is in contrast with those reported by other authors analyzing data in other countries such as India and China. They speculated that major improvements in air quality due to the lockdown were only found in places that had higher levels of air pollutants before the COVID-19 pandemic, compared to locations with relatively clean air to begin with such as in New York City in the US. We suggest that the improvement trend based on the air quality data from January to May (from boreal winter to autumn) in 2020 and in previous years in their work is due to the improvement in the emission (such as less solid fuel heater emission) and hence, masked the small improvement in 2020 due to the COVID-19 lockdown. A comparison of trend before and after the COVID-19 lockdown period in 2020 with those in previous years is not a reliable method as the change due to the seasonal change in meteorology and emission is larger than the change in emission due to the lockdown. A more complex trend analysis that takes into account the meteorology is to include other variables such as wind speed, wind direction, temperature, and humidity. Most authors such as Zangari et al. (2020) did not take into account the short term change in meteorology in the trend analysis. The results of our linear trend analysis show that trends in NO_2_, CO, PM_2.5_, and O_3_ before and after the lockdown can be due to the seasonal change in meteorology and emission in addition to the complex photochemical reaction between primary pollutants NO_2_, CO, and PM_2.5_ and secondary pollutants (O_3_ and secondary organic or inorganic aerosols components of PM_2.5_).

Our study shows that the linear regression trend method cannot be used to detect the effect of the short lockdown period (a few months) due to the change in emission using an interannual comparison of trend unless the intra-annual effect or seasonal meteorological change is taken into account. Huang et al. (2020) [12] in their study of the effect of the COVID-19 lockdown on air quality in Hong Kong, used the treatment and control method to compare the air pollutant concentration in each month of the lockdown period from February to April 2020 with the those in previous years of 2019, 2018, and 2017. Their results are similar to those of our study: The NO_2_, CO, and PM_2.5_ concentrations during the COVID-19 lockdown in 2020 are reduced but O_3_ increased and the concentrations of these pollutants when compared with those in January 2020 (before the COVID-19 lockdown) are inconclusive. They explained that the reason for the uncertainty is the seasonal change or meteorological difference between January and February to April. Our results show the importance of controlling the effect of meteorology or seasonal change. On the other hand, by estimating the change in emission from quantifying the change in traffic activities during and before the lockdown, the control of meteorology is achieved using the air quality model to predict the change in air quality due to the lockdown effect.

Wang, Chen et al. (2020) [20] used the WRF-CMAQ model to predict the effect of the COVID-19 lockdown in China on the PM_2.5_ pollution under reduced emission scenarios and showed that even though the levels in many cities are reduced due to the large emissions reduction in transportation and slight reduction in the industrial sector, that alone would not help avoid the severe air pollution, especially when meteorology is unfavorable. This corresponds with our WRF-CMAQ run during the lockdown meteorological condition and under the reduced motor vehicle emission of 30% but the decrease in PM_2.5_ level is insignificant. Marlier et al. (2020) [22] also used the WRF-CMAQ model to simulate air quality in 2019 and 2020 during the lockdown period. However, they use the same emission for both simulated scenarios and argue that the difference between the simulation and observations in the 2020 lockdown period suggests impacts from the reduced activity on air pollution, while comparisons between 2019 and 2020 indicate the influence of meteorology on driving air pollutant concentration changes with constant emissions. Their reason is only valid if the uncertainty in the model itself is not significant or smaller than the impact due to the reduced emission. Wang, Zhang et al. (2021) [21] used the WRF-CMAQ model to simulate the periods of prior lockdown, during lockdown, and post lockdown with the reduced emission during lockdown and normal emission in prior and post lockdown periods to determine the air quality change in the Pearl River Delta of China. The emission reduction is mainly from the transport sector. The WRF-CMAQ simulation approach using the reduced emission is similar to that in our study except that the meteorology changed in each period of their simulation. Their results showed a significant improvement in NO_2_ and PM_2.5_ concentration but O_3_ had no change during the lockdown. As meteorology was not controlled in their study, there is uncertainty in determining whether the improvement in air quality during the lockdown was from the emission reduction or from the favorable meteorological condition.

The results from the use of WRF-CMAQ air quality model in our study to predict the effect of COVID-19 lockdown on air quality in GMR of Sydney confirm and are consistent with the results from the statistical and diurnal analysis.

## 5. Conclusions

The reduction of emission mainly from the transport sector during the COVID-19 lockdown period in Sydney and NSW in April to June has resulted in an improvement of air quality in the GMR in terms of the reduction of the pollutants of NO_2_, CO, and PM_2.5_. However, this also caused an increase in the secondary pollutant O_3_ median and average levels. The simulation using the air quality model on the effect of emission change on air quality also confirmed the above results derived from the statistical and diurnal analysis of air quality monitoring data. Even though the change in air quality during the lockdown period is small, it is detectable.

The lesson from the COVID-19 lockdown showed that the improvement in air quality due to the reduction of transport emission is small but measurable. In addition, it highlights the importance of not only the transition from the current dominant combustion-technology vehicle fleet to the electric or non-combustion technology, but also the effort in emission reduction from other sources (shipping, aircraft, locomotive, power stations, etc.) including biogenic sources such as dust to make a significant impact on air quality improvement in the urban areas.

## Figures and Tables

**Table 1:** Summary of methods and data used in this study and in other referenced studies.

Method.	Analysis	Data	Other Studies	Data used in This Study
Control and Treatment method	Statistical (box plot, probability density distribution, summary statistics, etc.)	Monitoring data (temporal)	Rodríguez-Urregoa et al. (2020), Habibi et al. (2020), Ming et al. (2020), Chu et al. (2020), Brimblecombe et al. (2020), Huang et al. (2020), Sharma et al. (2020), Berman and Ebisu (2020), Brimblecombe et al. (2021), Ji and Tartarini (2020), Donzelli et al. (2021)	2016, 2017, 2018, 2019, and 2020 CO, NO_2_, PM_2.5_, and O_3_
		Remote sensing data (spatial)	Venter et al. (2020), Miyazaki et al. (2020), Li and Tartarini (2020), Marlier et al. (2020), Brimblecombe et al. (2021)	NO_2_ from OMI Aura satellite sensor and CO from MOPITT and MERRA
	Trend	Monitoring data	Zangari et al. (2020)	2016 to 2020 CO, NO_2_, PM_2.5_, and O3
	Diurnal	Monitoring data	Singh et al. (2020), Shi and Brasseur (2020), Jiang et al. (2021)	2016 to 2020 CO, NO_2_, PM_2.5_, and O_3_
Modelling method	WRF-CMAQ	Meteorological and emission data	Wang, Chen et al. (2020), Marlier et al. (2020), Zhao et al. (2020), Wang, Zhang et al. (2021)	NCEP reanalysis meteorological and EDGAR + local NSW emission inventory
	Other air quality models and methods		Sharma et al. (2020) (WRF-AERMOD), Miyazaki et al. (2020) (Tropospheric Chemistry Reanalysis version 2 or TCR-2). Wang, Wen et al. (2020) Machine learning method	

**Table 2:** Traffic counting site and comparison of 2019 and 2020 median hourly traffic volume.

Traffic Counting Site	Location (Lat, Lon) in Degrees	Nearest Monitoring Site	2019 (Median, Mean) Hourly Traffic Volume	2020 (Median, Mean) Hourly Traffic Volume	Percentage Decrease
Liverpool Road	(−33.887, 151.073)	Chullora	(2830, 2421)	(1968, 1902)	30%
Hume Highway	(−33.905, 151.041)	Chullora	(3476, 3052)	(2801, 2667)	19%
Memorial Drive	(−34.384, 150.900)	Wollongong	(1613, 1669) *	(896, 1076)	44%
Donald Street	(−32.918, 151.740)	Newcastle	(1284, 1355)	(822, 1113)	36%
Pacific Highway	(−32.818, 151.692)	Beresfield	(1874, 1907)	(1266, 1555)	32%

(*) Data for 2019 is not available; data for 2018 is used instead.

**Table 3:** Summary statistics of NO_2_, CO, PM_2.5_, and O_3_ for the period from April to June in 2017, 2018, 2019, and 2020.

		NO_2_	CO	PM_2.5_	O_3_
2016	Mean	0.947	0.294	10.07	1.47
	Median	0.685	0.242	7.12	1.49
	Max	5.41	2.446	320.74	8.45
2017	Mean	0.944	0.234	7.437	1.27
	Median	0.736	0.191	6.503	1.15
	Max	6.128	2.107	51.97	5.24
2018	Mean	0.909	0.231	9.389	1.64
	Median	0.657	0.187	7.135	1.78
	Max	6.185	2.283	335.40	7.44
2019	Mean	0.912	0.211	8.790	1.34
	Median	0.694	0.181	6.694	1.29
	Max	5.301	2.785	295.25	5.77
2020	Mean	0.792	0.193	7.10	1.38
	Median	0.570	0.162	5.87	1.42
	Max	4.821	14.397	115.72	6.27
Welch *t*-test (2020 vs. 2019)		T = 20.615 *p*-value < 2.2 × 10^−16^ Different (null hypothesis rejected)	T = 8.36 *p*-value < 2.2 × 10^−16^ Different (null hypothesis rejected)	T = 25.121 *p*-value < 2.2 × 10^−16^ Different (null hypothesis rejected)	T = −4.93 *p*-value = 8.19 × 10^−7^ Different (null hypothesis rejected)

## Data Availability

Not applicable.
